# Polydimethylsiloxane/Nanodiamond Composite Sponge for Enhanced Mechanical or Wettability Performance

**DOI:** 10.3390/polym11060948

**Published:** 2019-06-01

**Authors:** Xuxin Zhao, Tao Wang, Yaoyao Li, Lei Huang, Stephan Handschuh-Wang

**Affiliations:** 1 College of Chemistry and Environmental Engineering, Shenzhen University, Shenzhen 518060, China; zhaoxx@szu.edu.cn (X.Z.); 2151110214@email.szu.edu.cn (Y.L.); 2 Functional Thin Films Research Center, Shenzhen Institutes of Advanced Technology, Chinese Academy of Sciences, Shenzhen 518055, China; tao.wang1@siat.ac.cn (T.W.); lei.huang@siat.ac.cn (L.H.)

**Keywords:** nanodiamond, nanocomposite, mechanical properties, surface wettability, infrared (IR) spectroscopy, oil/water separation

## Abstract

Polydimethylsiloxane (PDMS) is widely utilized in material science, chemical engineering, and environmental science due to its excellent properties. By utilizing fillers, so-called composite materials can be obtained with enhanced mechanical, wettability, or thermal conductivity performance. Here, we present a simple, cost-effective approach to vary either the mechanical properties (Young’s modulus) or surface wettability of bulk PDMS and PDMS sponges simply by adding nanodiamond filler with different surface terminations, either oxidized (oND) or hydrogenated (reduced, rND) nanodiamond. Minuscule amounts of oxidized nanodiamond particles as filler showed to benefit the compressive Young’s modulus of composite sponges with up to a 52% increase in its value, while the wettability of composite sponges was unaffected. In contrast, adding reduced nanodiamond particles to PDMS yielded inclined water contact angles on the PDMS/nanodiamond composite sponges. Finally, we show that the PDMS/rND composites are readily utilized as an absorbent for oil/water separation problems. This signifies that the surface termination of the ND particle has a crucial effect on the performance of the composite.

## 1. Introduction

In science and industrial applications, polymer nanocomposites have received increasing attention over the last several years. This can be related to the remarkable improvements offered by the incorporation of nano-sized fillers, such as fullerenes [1], carbon nanotubes [2,3,4], nanodiamond (ND) [5,6], inorganic nanoparticles [7], and graphene sheets [8,9,10]. Improvements to material properties often include improvements to the mechanical [11], thermal [11,12], and barrier properties [13,14,15] of the nanocomposite. Most of these fillers, however, are expensive, and surface functionalization has to be executed prior to their usage.

Recently, macroporous polymer substrates have emerged as a new research field, and great effort is currently being employed to tune these macroporous polymer substrates (also known as sponges) to the needs of the envisaged applications [16], such as liquid transport [17], catalysis [18], oil–water separation [19,20], flexible conductors [21,22], and sensors [23]. Polydimethylsiloxane (PDMS) sponges are such macroporous polymer substrates. For PDMS sponges, the important parameters are the fatigue resistance, mechanical properties, wettability, pore size, porosity, and pore interconnectivity. Of course, typical parameters such as the curing time, curing temperature, and prepolymer-to-cure ratio contribute to the mechanical properties. Pore size and porosity, however, exert an influence on the Young’s modulus of PDMS sponges as well. A typical improvement via the usage of nanoparticles is the incline of mechanical properties. Not limited to mechanical properties, other parameters can be changed by employing nanoparticles. For example, the wettability of melamine–formaldehyde sponges was altered by Gao et al. by decorating them with MoS_2_ nanosheets, yielding superhydrophobic and superoleophilic MoS_2_ sponges (SMSs) [24]. Therefore, it is of utmost importance to design the PDMS/filler composition to the needs of an appliance.

A versatile filler is single-digit-sized nanodiamond (ND) [25]. ND particles exhibit in their pristine state essentially the excellent properties of bulk diamond, including high thermal conductivity, electrical resistivity, superior hardness and Young’s modulus, biocompatibility, and chemical inertness even in harsh environments [26]. Therefore, ND particles find applications as micro-abrasion additives, in medicine (drug delivery, skin care, enterosorbent), suppression of dendrides in batteries, electrolytic and electroless metal plating (as an additive), heat transfer, magnetic resonance imaging, chromatography, proteomics, mass spectrometry, and catalysis [12,27,28,29]. Furthermore, as-produced ND particles offer a broad range of surface groups, such as carboxylic acid, ketone, aldehyde, amine, alcohol, and alkyl [26], offering plenty of options for surface modification. Alteration of the surface termination may be useful for improvement of the dispersion of the ND particles or for a mechanical improvement effect on the polymer nanocomposite [30]. For example, surface modification of ND with vinyltrimethoxysilane (VTS) was beneficial for improving suspension stability in a hydrophobic medium [31], while it also afforded an incline in the Young’s modulus of 25% at 0.2 wt % of silanized ND [30]. Notably, diverse surface termination originating from the fabrication method itself, the varying purification procedures, and post-processing, such as hydrogen annealing, yield contradictory results regarding the properties of the ND–polymer composites. The reinforcement of polymers is complicated by the size and shape of the filler [32]. Therefore, it is essential to ascertain the surface termination, size, and shape of ND particles prior to the actual experiment. 

In this article, we show that the surface termination of the ND particles is of pivotal importance for the effect on bulk PDMS and its porous structures. Particularly, change in the Young’s modulus and wettability variations of PDMS/ND composite result with increasing wt % of two different kinds—oxidized (oND) and hydrogenated (reduced, rND)—of nanodiamond particles blended in the PDMS prior to curing. Subsequently, this different behavior of composites based on oxidized or reduced ND particles is analyzed by FTIR measurements to find the cause for the diverging behavior of the two different composites. Furthermore, out of both composites, porous sponges were fabricated, and both the compressive modulus and wettability are assessed. As the mechanical properties of PDMS sponges and bulk material can be changed, likewise, such nanocomposites may find applications in flexible and wearable electronics, in environmental engineering, and as sensors. In contrast, PDMS/rND composite sponges with an inclined water contact angle show great potential as an absorption material for oil spills and are therefore an interesting candidate for oil/water separation problems. Therefore, applicability towards a specific field may be enhanced by traits inherited by the different kinds of nanofillers.

## 2. Materials and Methods

### 2.1. Materials

The oxidized ND (oND) particles utilized in this article are commercially available detonation ND particles bought as a powder from Tongli (P. R. China). The diameter according to the producer was 4–6 nm. The reduced ND (rND) particles are commercially available detonation ND particles, which were post-processed by the producer (New Metals and Chemicals Corporation, Ltd., Tokyo Japan). The rND particles (NanoAmando) were purchased as an aqueous colloidal solution with a weight percentage of 2.5. The diameter according to the producer is 3 nm. PDMS (Sylgard 184) was supplied by Dow Corning (Midland, Michigan, United States). The solvent ethanol (0.79 gcm^−3^, 99.7%) was bought from Aladdin (Shanghai, China). Water, if referred to in this article, denotes purified deionized (DI) water, i.e., in water contact angle measurements and preparation of the sponges.

### 2.2. Fabrication of the PDMS/oND Composite

PDMS prepolymer (component A) was poured into a plastic cup and a certain amount (which denotes the desired wt %) of oxidized ND particles was added. The wt % for the oND was between 0.01 and 0.5 wt %. Afterwards, the composite was mixed extensively with a hand mixer. After the filler was agitated by ultrasonication for 30 min to disperse the oND particles, the cure (component B) was added in a weight ratio of 10:1, according to the supplier’s instruction. The composite of PDMS prepolymer, cure, and oND particles was mixed for 5 min, followed by a second ultrasonication step for 30 min, and finally, cured for 3 h at 65 °C.

### 2.3. Fabrication of the PDMS/rND Composite

Firstly, a certain volume (which denotes for the desired wt % in the PDMS composite) of aqueous solution of reduced ND particles was poured into a glass beaker and the water was removed. The resulting solid was dissolved in 5 mL ethanol and agitated ultrasonically for around 2 h. PDMS prepolymer (component A) was poured into a plastic cup, and 5 mL of the previously prepared rND colloidal solution in ethanol was added. The mixture was extensively mixed and subsequently agitated by ultrasonication for 30 min. Afterwards, the cure (component B) was added to the slurry, mixed, and agitated again. Finally, it was placed in an oven at 65 °C for 3 h.

### 2.4. Fabrication of the PDMS/ND Composite Sponges

PDMS composite sponges were prepared via a method similar to that detailed in our previous publications on Ecoflex sponges for oil–water separation and PDMS sponges for flexible electrical conductors by the sugar leaching technique [17]. Briefly, the slurry (see *Fabrication of PDMS/rND composite* or *Fabrication of PDMS/oND composite*) was filled into a plastic cup, commercially available sugar cubes were immersed into the slurry, and reduced pressure was applied to infiltrate the voids of the sugar cubes with the slurry. Air pockets were also removed by the reduced pressure. Consequently, the PDMS/sugar/ND composite was cured at 65 °C for 3 h. Afterwards, the sugar cubes were cut, followed by removal of the sugar template in water for 3 h. Finally, the PDMS sponge was washed several times with ethanol.

### 2.5. Measurement of Mechanical Parameters

For tensile (Young’s modulus) measurements, the composite was poured prior to curing into a tensile test mold with the following approximate parameters: length 28 mm, width 3.9 mm, and height 3.9 mm. The Young’s modulus was measured using a universal mechanical testing machine (Autograph AG-X plus 100N, Schimadzu, Kyoto, Japan) with a measurement velocity of 20 mm/min. The compressive modulus was determined using the same machine at a measurement velocity of 5 mm/min. Typical dimensions of a PDMS sponge were length 17 mm, width 17 mm, and height 9 mm. The maximum compressive strain was set to 50%. 

### 2.6. Analysis of the Raw Materials, Composite Sponges, and Bulk Composite

An FTIR Spectrometer (Bruker Vertex 70, Billerica, Billerica, United States) was utilized to ascertain the surface termination of ND particles and to analyze the effect of rND and oND particles on the PDMS. The particle size distribution of the ND particles in their colloidal solution was assessed using a Zetasizer Nano ZS (Malvern Instruments) with a laser wavelength of 633 nm via dynamic light scattering. Square quartz cuvettes were used for determination of the hydrodynamic diameter (*d*_h_), whereas for Zeta potential measurements, disposable folded capillary cells (DTS 1070, Malvern instruments) were used. Contact angle microscopy (SDC-200, Sindin, Dongwan, China) was employed as a means to determine the wettability of the sponges.

## 3. Results and Discussion

### 3.1. Analysis of Oxidized and Hydrogenated ND Particles

**Figure 1:**
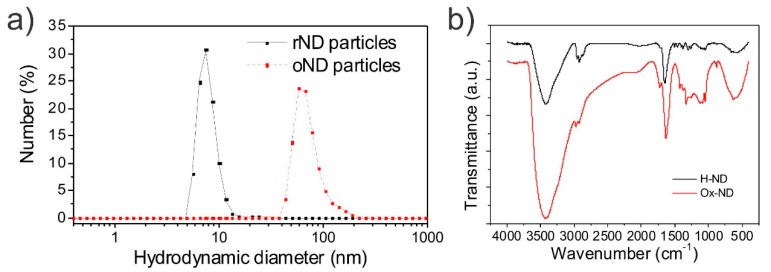
(**a**) Number weighted size (hydrodynamic diameter) distribution determined by DLS of hydrogenated (reduced) nanodiamond (rND) and oxidized ND (oND); (**b**) Fourier transform infrared spectra (FTIR) of as-received reduced and oxidized ND particles.

ND particles feature an extreme surface-to-volume ratio [33] and thus, the properties in composite materials and their reactivity are strongly dependent on the surface termination of the ND particles. In this study, we focus on the difference between reduced (hydrogen annealed, rND) ND particles and oxidized (as-made, oND) detonation ND particles. However, it should be noted that the surface modification of ND particles is a broad and fertile means to vary diverse properties of polymer/ND composites [33,34,35]. Reduced diamond surfaces and rND particles feature a hydrogen termination and are hydrophobic [36]. The size of the rND particles according to the supplier is 4.1 ± 0.7 nm. By dynamic light scattering (DLS), the hydrodynamic diameter (*d*_h_) is accessible, which is slightly larger than the diameter of the particle itself. DLS revealed that, indeed, rND particles dispersed in water at pH ≈ 7 possess a single-digit nanometer size (see Figure 1a), attributable to the high Zeta potential (*ζ*) of the rND particles (*ζ* ≥ 30 mV) [37]. This accounts for the good colloidal stability of the rND particles in aqueous solution and may help during the dispersion in polymers. On the other hand, oxidized ND particles possess, according to the producer, a size of 4–6 nm. However, only double-digit nanometer sized particles could be observed in DLS measurements at pH ≈ 7. This is related to the low Zeta potential of the oxidized particles (−30 mV ≤ *ζ* ≤ +30 mV) [38], resulting in low colloidal stability [39]. The surface of oND particle is covered with diverse functional groups, ranging across OH, CO (ketones), carboxylic acids, amines, etc. [26,40]. Due to this surface termination, oxidized diamond surfaces and oND particles are hydrophilic [36].

The different surface terminations of oxidized and reduced ND particles can be observed in the IR spectra measured via FTIR spectroscopy. oND features a broad and strong absorption band denoting the stretching vibrations of hydroxyl and amino groups at 3432 cm^−1^ and 3429 cm^−1^, respectively (see Figure 1b). The absorption peaks at 2970 cm^−1^ and 2928 cm^−1^ signify the asymmetric and symmetric C–H vibration. Furthermore, carbonyl and carboxyl moieties were identified by the presence of an absorption band at 1723 cm^−1^. The broad absorption peak at 1630 cm^−1^ and the shoulder at 1590 cm^−1^ denote O–H and N–H vibration of adsorbed surface water and amino moieties located at the interface of the oND particle. Finally, in the fingerprint range (between 1500 and 1000 cm^−1^), oND particles feature a broad palette of absorption bands, which relate to O–H deformation, C–O–C stretch, epoxy C–O stretch, C–C stretch, amide C–N stretch and C–N–H deformational vibrations, and many other groups [26]. In contrast, the fingerprint region in particular differs for rND particles with a few weak and narrow absorption bands. Furthermore, a shoulder at 1590 cm^−1^ cannot be detected while the peaks at 1650 cm^−1^ (compare oND at 1630 cm^−1^) and 3426 cm^−1^ are weaker than for oND. Finally, the absorption peak denoting C=O bonds (1730 cm^−1^), such as carboxyl, aldehyde, ketone, etc., is barely visible in the spectrum of rND. However, broad absorption peaks at 3426 cm^−1^ and 1650 cm^−1^ remain, which are related to the absorption of OH from water adsorbed to the surface of the rND particles [41].

### 3.2. Mechanical Properties of PDMS/ND Composites

**Figure 2:**
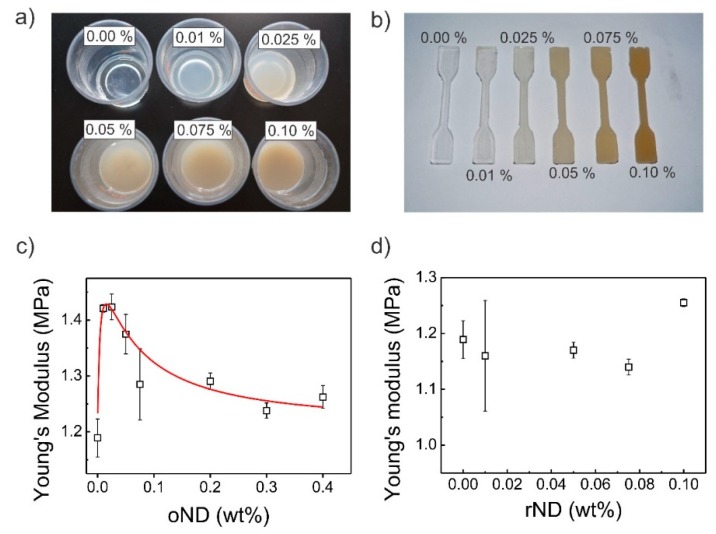
(**a**) Optical images of bulk polydimethylsiloxane (PDMS)/oND composite before curing and (**b**) after curing in a standardized mold. The percentage numbers denote the weight percentage of oxidized nanodiamond in the nanocomposite. Young’s moduli of bulk PDMS/ND composites with increasing ND content. (**c**) Oxidized hydrophilic ND particles and (**d**) reduced hydrophobic nanoparticles were blended into the PDMS prepolymer prior to curing.

The mechanical properties of PDMS itself are dependent on several fabrication parameters, including the prepolymer-to-cure weight ratio, curing time, and curing temperature [42,43]. Moreover, the mechanical properties of PDMS can also be changed by blending it with other silane-based and curable polymers, such as Ecoflex [44]. In this article, these fabrication parameters, however, are maintained at 10:1 (prepolymer-to-cure ratio), 3 h curing time, and 65 °C curing temperature. Here, we focus on the change of the Young’s modulus with two different kinds of ND particles, either oxidized or reduced ND particles, and the change upon increasing ND particle concentration. Gogotsi and Voznyakovskii suggested that the addition of a low amount of ND particles enhances the mechanical properties of PDMS [33,45]. Therefore, bulk PDMS/ND composites with oND and rND particles were prepared. The uncured PDMS/oND composite is illustrated in Figure 2a, while the cured is illustrated in Figure 2b. Pristine PDMS is transparent, while both kinds of PDMS/ND composites appear milky at first and grey/brown with high ND concentration. Furthermore, in the optical images, the dispersion of the nanoparticles appears homogeneous, which is advantageous for the improvement of mechanical parameters. Indeed, the addition of oND particles to PDMS prior to curing yields in increased Young’s modulus, as illustrated in Figure 2c (see also stress–strain graphs in the supporting information). However, the Young’s modulus is not monotonically increasing; rather, it passes through a global maximum (1.43 ± 0.02 MPa) between 0.01 wt % and 0.025 wt %, where the modulus is around 20% higher than the modulus of PDMS itself. This incline is comparable to the enhanced Young’s modulus, attributed to increased crosslinking density, obtained by the addition of 0.2 wt % silanized ND particles [30]. Afterwards, the Young’s modulus decreases with increasing wt % of oND particles to around 1.25 ± 0.02 MPa, which is still higher than that of pristine PDMS (1.19 ± 0.04 MPa). This behavior suggests at least two concurrent effects: one process increases the Young’s modulus while the second decreases the Youngs’s modulus. Several authors, who have engaged with polymer reinforcement by nanoparticles, have suggested that a reduction of the Young’s modulus is related to chain distortions, and this effect gets more drastic with increasing aggregation of the nanoparticles [45], while a reinforcing effect may be related to polar interactions or bond formation induced by or with the oND particles (see FTIR analysis).

On the other hand, PDMS/rND composite were only measured between 0 and 0.1 wt % as the beneficial concentration for the Young’s modulus of oND particles was ascertained to be around 0.025 wt % and due to the high cost of them. The addition of rND particles to PDMS yielded no enhancement of the Young’s modulus (see also the stress–strain graphs in Figure S2). Moreover, the Young’s modulus is slightly lower at 1.14 ± 0.04 MPa than that of pristine PDMS (see Figure 2d). The slightly lower Young’s modulus may originate from the solvent added in the fabrication step and/or from the unprofitable effect of chain distortion, which are alleviated presently. A reinforcement effect of rND on the polymer similar to that of oND particles was not observed due to the chemically inert surface termination of the rND particles. With this knowledge at hand, the effect increasing the Young’s modulus can be ascribed to the surface termination of the ND particle as it is only present for oND particles. The reduction in Young’s modulus with increasing ND filler concentration is present for both ND particle types and is therefore related to the ND particles themselves. The strength of the latter effect is lower for rND particles than for oND, which can be explained by two factors. Firstly, the oND particles tend to aggregate and are bigger than the rND particles (see previous section, DLS). With a higher concentration, this aggregation is more pronounced, which yields a lower Young’s modulus [46]. In contrast, rND particles feature inclined colloidal stability and, consequently, no aggregation should be present. Secondly, rND particles are hydrophobic and appear to be very surface active as they increased the bubble stability during vacuum treatment and gas removal during the PDMS/rND composite sponge fabrication. This suggests that the rND particles favor to adsorb at the PDMS/gas interface. This coincides with an incline in water contact angle from 112° for pristine bulk PDMS to 118° for bulk PDMS/rND (see Figure S3). A change in the water contact angle of the PDMS/oND composite, however, could not be observed (see also contact angles on sponges).

**Figure 3:**
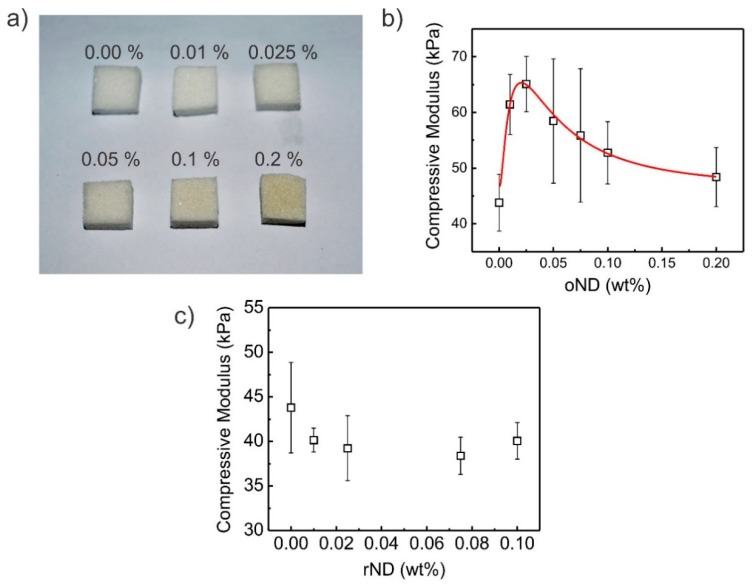
(**a**) Optical images of PDMS/oND composite sponges. Compression moduli of PDMS/ND composite sponges with increasing wt % of blended ND particles. (**b**) Oxidized ND particles and (**c)** reduced ND particles were used.

Similar to the bulk PDMS and bulk PDMS/ND composites, composite sponges were prepared by the sugar leaching technique. The sponges are shown in Figure 3a. PDMS sponges appear white due to the refraction of light at the copious arbitrary interfaces inside of it. With high oND particle concentration, the PDMS/oND composite sponge is tinged grey/brown, similar to the as-made bulk material, though not as pronounced (see Figure 3a). The pore size (radius) of the PDMS sponges was measured by confocal laser scanning microscopy to be around 50 µm, as shown in Figures S4 and S5, while the pore size can be altered by applying tensile strain. The pore size in leaching approaches is dependent on the grain size of the sacrificial template (here sugar particles) and it therefore remains constant for both types of composite sponges. Another important parameter of PDMS sponges is the compressive modulus, especially for application as pressure sensors. The compressive moduli of PDMS/oND composite sponges and the previously discussed Young’s moduli of PDMS/oND bulk composites follow a similar trend. For your evaluation, three compressive stress–strain graphs are attached in the supporting information (Figure S6). The global maximum, however, is shifted to a concentration of 0.025 wt % oND. At 65 ± 5 kPa, the global maximum is 52% higher than that of pristine PDMS sponges, which possess a compressive modulus of 43 ± 5 kPa. After this global maximum, the modulus gradually decreases with increasing wt % of oND particles. At the highest concentration measured in this study (0.2 wt %), the compressive modulus was determined to be 48 ± 5 kPa, still 11% higher than the compressive modulus of pristine PDMS. Certainly, the trend of the curve can be explained similarly to the bulk PDMS/oND composite by a competition of the fortification and weakening of the modulus by polar forces and bond formation and by chain distortions, respectively. In contrast to this result, the compressive modulus of PDMS blended with rND particles dwindles with increasing rND particle concentration to 38 ± 2 kPa at a concentration of 0.075 wt % rND particles—a reduction of 12% compared to that of pristine PDMS sponges (see also Figure S7).

### 3.3. FTIR Analysis of PDMS/ND Composites

**Figure 4:**
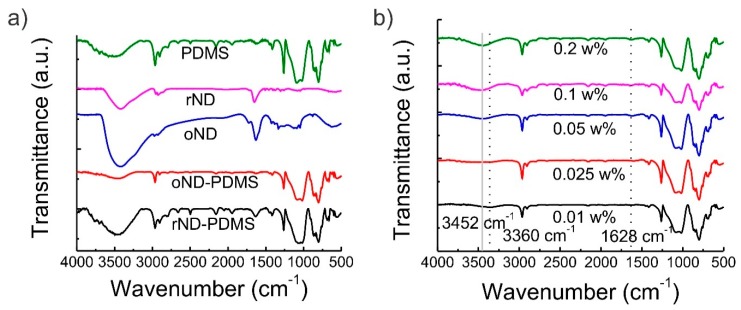
(**a**) FTIR transmission spectra of PDMS (green), reduced (H-terminated) ND (purple), oxidized ND (blue), PDMS/oND composite with 0.1 wt % oND, and PDMS/rND composite with 0.1 wt % rND. (**b**) FTIR transmission spectra of PDMS/oND particles dependent on the concentration of the oND particles. The concentration ranged between 0.01 and 0.2 wt %.

To shed light on the mechanism of mechanical reinforcement of PDMS/oND composites, FTIR spectroscopy was utilized. The FTIR spectra of PDMS (after curing), reduced (H-terminated) ND, oxidized ND, PDMS/oND composite, and PDMS/rND composite are shown in Figure 4a. The spectrum of PDMS features distinct absorption bands in the fingerprint region. For example, the peak at 560 cm^−1^ denotes SiO_2_ defects [47], methyl rocking and SiC vibrations appear around 802 cm^−1^, the bending vibration of Si–H at 913 cm^−1^ signifies residual cure (crosslinker) [48], and the broad absorption denoting the stretching vibrations of Si–O–Si of the oligomer is located at 1013 and 1080 cm^−1^ [49]. At higher wavenumbers, the symmetric (*ν*_s_(CH_3_) ≈ 2963 cm^−1^) and asymmetric (*ν*_a_(CH_3_) ≈ 2905 cm^−1^) stretching vibration of sp3 hybridized methyl groups can be found, while the deformation vibration is located at 1260 cm^−1^. The weak broad band absorptions centered around 3460 cm^−1^ and 3720 cm^−1^ signify the presence of –OH groups, which can be ascribed to both adsorbed surface water and free SiOH groups (peak broadening due to hydrogen bonding) [47,50,51]. While the spectrum for the PDMS/rND composite looks rather similar to the spectrum of pristine PDMS, a clear difference can be seen between pristine PDMS and the PDMS/oND composite. The spectrum of the composite features only a very weak absorption band related to adsorbed water (3460 cm^−1^), while the absorption band at 3720 cm^−1^ virtually vanished. This signifies a reaction of the silanol groups with either amine or carboxyl moieties of the oND surface. A further lead for this reaction is the minuscule absorption band at 1628 cm^−1^, which signifies the absence of OH groups in the composite, and the absence of the carboxylic acid ν(CO) at 1723 cm^−1^, implicating the participation of DND-bound COOH groups as grafting sites for the PDMS [52]. The absence of the latter may be attributed to the minuscule concentration (0.1 wt %) of the oND particles in the composite. Figure 4b shows the changes in the FTIR spectra of PDMS/oND composites with increasing oND concentration. At the lowest concentration (0.01 wt %), the absorbance band around 3452 cm^−1^, denoting the OH vibration, is barely visible, while a weak absorption band at 3360 cm^−1^ can be observed. Furthermore, the absorption band at 1628 cm^−1^ cannot be observed either. With increasing oND concentration, a blue-shift of the absorption band at 3360 cm^−1^ to 3452 cm^−1^ can be observed. Concomitantly, absorption bands at 1628 cm^−1^ and 1780 cm^−1^ (ester) arise (see Figure S8). These absorption bands get stronger with increasing oND concentration. These results imply that at first, residual silanol groups of PDMS are used to covalently bind oND particles. At low oND concentrations, this may lead to crosslinking of PDMS via these oND particles, which increases the Young’s modulus. By further increasing the oND particle concentration (˃0.025 wt %), most silanol groups are used and the hydroxyl groups of the oND appear visible in the IR spectra (carboxylic acid). This coincides with the decline of mechanical properties at oND concentrations higher than 0.025 wt %. Therefore, we conclude that most of the silanol groups are already occupied/bounded and further addition just yields the negative effects of the nanofiller on the polymer, such as chain distortion.

### 3.4. Surface Wettability of the As-Made PDMS/ND Composite Sponges

**Figure 5:**
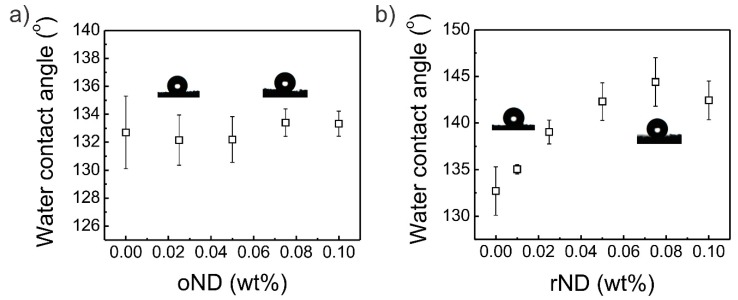
Water contact angle on PDMS/ND composite sponges dependent on the concentration of (**a**) oxidized and (**b**) reduced ND particles. The error bars denote the standard deviation of at least 3 single measurements on the PDMS sponges.

In contrast to the measurement of the Young’s modulus, the wettability of PDMS sponges is not altered by the addition of oxidized ND particles. Rather, the water contact angle on PDMS/oND composite sponges remains virtually constant at around 132°, which is consistent with the water contact angles measured on pristine PDMS sponges [53]. This means that the oxidized nanodiamond particles do not exert an effect on the wettability properties of the composite sponge, even though the oND particles are known to be hydrophilic and, intuitively, this suggests that the water contact angle will be diminished. This effect can be ascribed to the chain mobility of PDMS and its ability for hydrophobic recovery, i.e., after oxidation with oxygen plasma, PDMS quickly recovers its hydrophobicity due to directed chain movement. On the other hand, the addition of reduced nanodiamond particles as a filler yields an incline in the water contact angle, as shown in Figure 5b. The contact angle increases quite linearly until a value of around 144° is achieved at a concentration of 0.075 wt % rND. Afterwards, the water contact angle stagnates, which we assign to the fact that the adsorption of rND particles to the surface of the PDMS sponge stagnates. With this result, we substantiate the claim that a proper choice of PDMS/ND composite sponge is of pivotal importance for its performance in any application. Finally, the as-made PDMS/rND composite sponges (0.025 wt % rND) were employed as an absorption material in an oil/water separation problem, as shown in Figures S9 and S10. The composite sponges absorbed paraffin oil (dyed red) rapidly up to an amount of 1.8 g paraffin oil (see also Figure S11). After three short cleaning steps (10 min each while stirring) with the composite sponges, no oil phase was visible, signifying that the composite sponge with enhanced wettability is a promising material for oil/water separation processes.

## 4. Conclusions

The effect of oxidized and reduced nanodiamond particles on PMDS bulk material and PDMS sponges was investigated. Oxidized nanodiamond particles showed to improve the Young’s modulus of bulk PDMS and porous (sponge-like) PDMS by 20 and 52% (around 0.025 wt % of oND), respectively, while reduced nanodiamond particles showed to weaken the mechanical properties of PDMS slightly. This weakening was attributed to chain distortions, which apparently are also present at high concentrations of oxidized nanodiamond particles. However, at the beneficial concentrations (0.01 and 0.025 wt %) the reinforcement effect of oND particles predominates the mechanical properties of the PDMS/oND composite. Notably, the concentration of nanofiller and its consumption is low, reducing production costs compared to other fillers. The reinforcement of this composite was explained by the crosslinking of PDMS macrochains via the carboxylic acid groups of the oND particles. The surface wettability was found to be virtually unchanged when using oND particles. In contrast, when employing rND particles, the water contact angle inclined from around 132° to 145°. This signifies that PDMS sponges can be tailormade to the need of the envisaged application, such as pressure sensors (change in Young’s modulus, oND). In contrast, the enhanced wettability (rND) of composite sponges was beneficial for oil/water separation. This study shows that the knowledge of the surface termination of nanoparticles—in this case, nanodiamond—is of pivotal importance to the effect of the nanofiller and the resulting field of application of the fabricated nanocomposite.

## Supplementary Materials

The following are available online at https://www.mdpi.com/2073-4360/11/6/948/s1, Figure S1 Stress-strain graph used to determine Young’s modulus of pure PDMS and PDMS/oxidized ND. Figure S2 Stress-strain graph used to determine Young’s modulus of pure PDMS and PDMS/reduced ND composites. Figure S3 Water contact angle on bulk PDMS/rND (left) and pristine PDMS (right). Figure S4 (a) and (b): Microscopy images of the sponge (3.5 mm) at 10xand 30x magnification, respectively, (c) confocal fluorescence images of the PDMS sponge stained with Rhodamine 6G, (d) SEM micrographs of the PDMS sponge (scale bar 1.5 mm) and (e) images of the water contact angle experiment on the PDMS sponge for tensile strain ranging from −50% to +400%. Figure S5 Pore size as determined by CLSM dependent on the tensile strain. Figure S6 Compressive stress-strain graph for determination of compressive modulus of PDMS sponges and PDMS/oND composite sponges. Figure S7 Compressive stress-strain graph for determination of compressive modulus of PDMS sponges and PDMS/rND composite sponges. Figure S8 Zoom in of FTIR spectra (main Manuscript, Figure 4), signifying change in absorption bands 1780 and 1628 cm-1. Figure S9 Separation of a paraffin oil/water emulsion in three steps with a PDMS/rND composite (0.025 wt % rND) sponges. Figure S10. Microscopy images of the oil (paraffin) in water emulsion after the 2nd and 3rd cleaning step, as depicted in Figure S9. Figure S11. Relative absorbed mass of acetone, dichloromethane and paraffin oil by the rND-PDMS composite sponges (0.05 wt %).
