# Supplementary material for: Polydimethylsiloxane/Nanodiamond Composite Sponge for Enhanced Mechanical or Wettability Performance
Source: Polymers (Basel). 2019 Jun 1;11(6):948. doi: 10.3390/polym11060948 (PMC6631953; doi:10.3390/polym11060948)
Supplement: Supplementary file 1 [file polymers-11-00948-s001.pdf]

Polydimethylsiloxane/Nanodiamond Composite Sponge for Enhanced Mechanical or Wettability Performance

Xuxin Zhao[a], Tao Wang[b], Yaoyao Li[a], Lei Huang[b] and Stephan Handschuh-Wang[a,*]

[a]College of Chemistry and Environmental Engineering, Shenzhen University, Shenzhen 518060, China

[b]Functional Thin Films Research Center, Shenzhen Institutes of Advanced Technology, Chinese Academy of Sciences, Shenzhen 518055, China

*Corresponding authors: Stephan Handschuh-Wang, Email: stephan@szu.edu.cn

Supporting Information

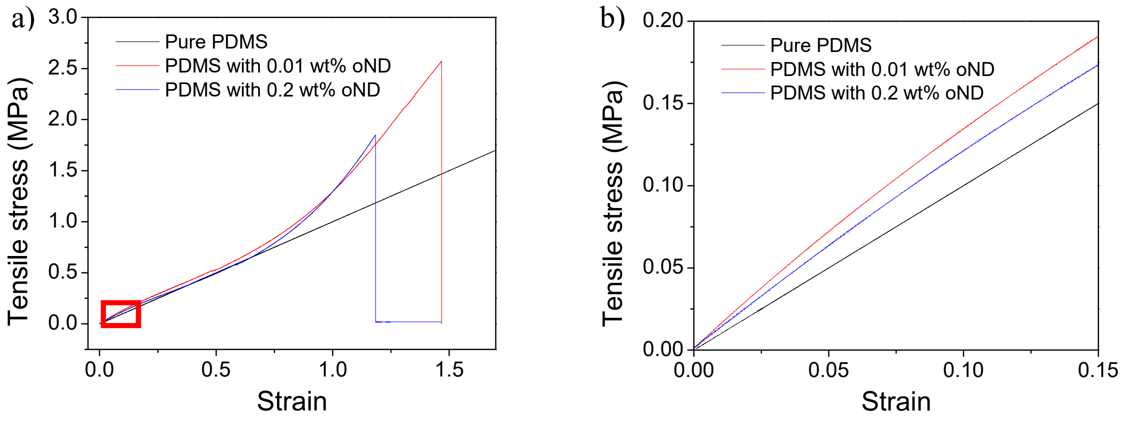

Figure S1 Stress–strain graph used to determine the Young's modulus of pure PDMS and PDMS/oxidized ND composites (a) and its magnification (b) at the location specified with a red rectangle in the graph of (a). The *x* axis was adjusted by an *x*-axis offset for better comparison.

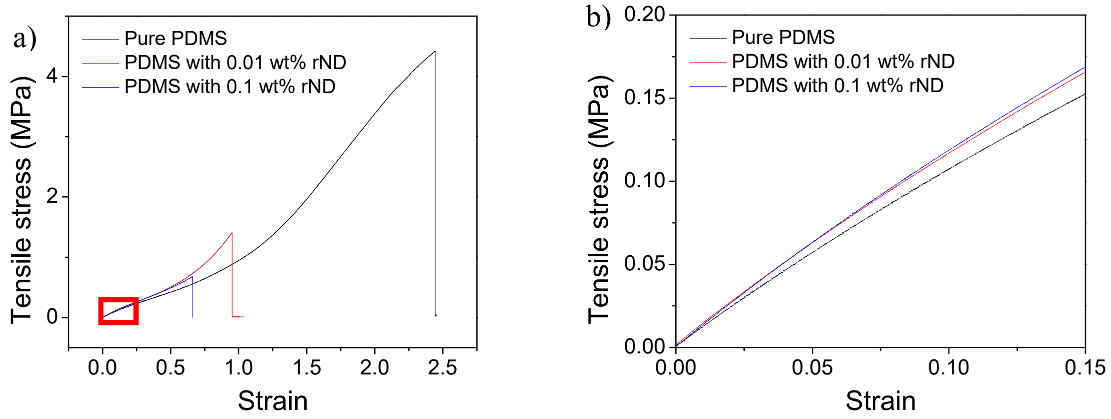

Figure S2 Stress–strain graph used to determine the Young's modulus of pure PDMS and PDMS/reduced ND composites (a) and its magnification (b) at the location specified with a red rectangle in the graph of (a).

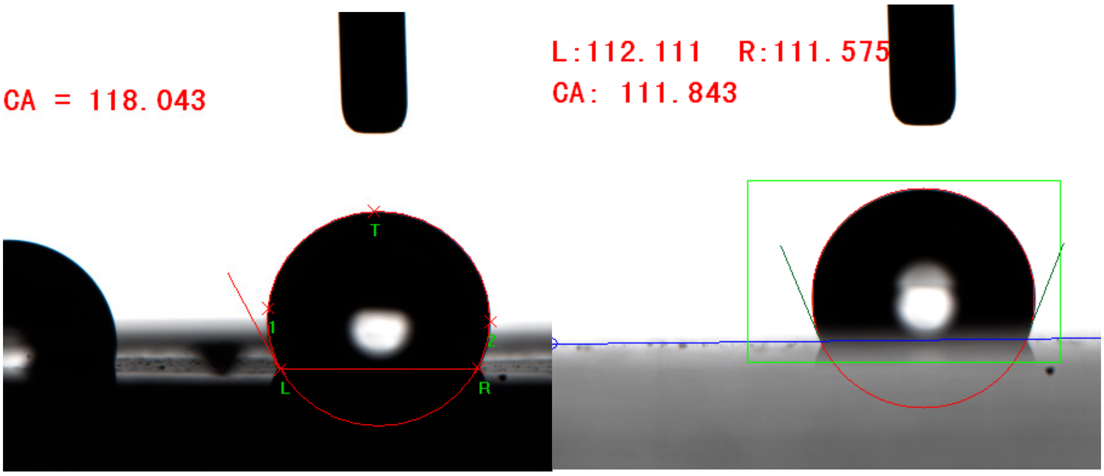

Figure S3 Water contact angle on bulk PDMS/rND (left) and pristine PDMS (right).

Pore size:

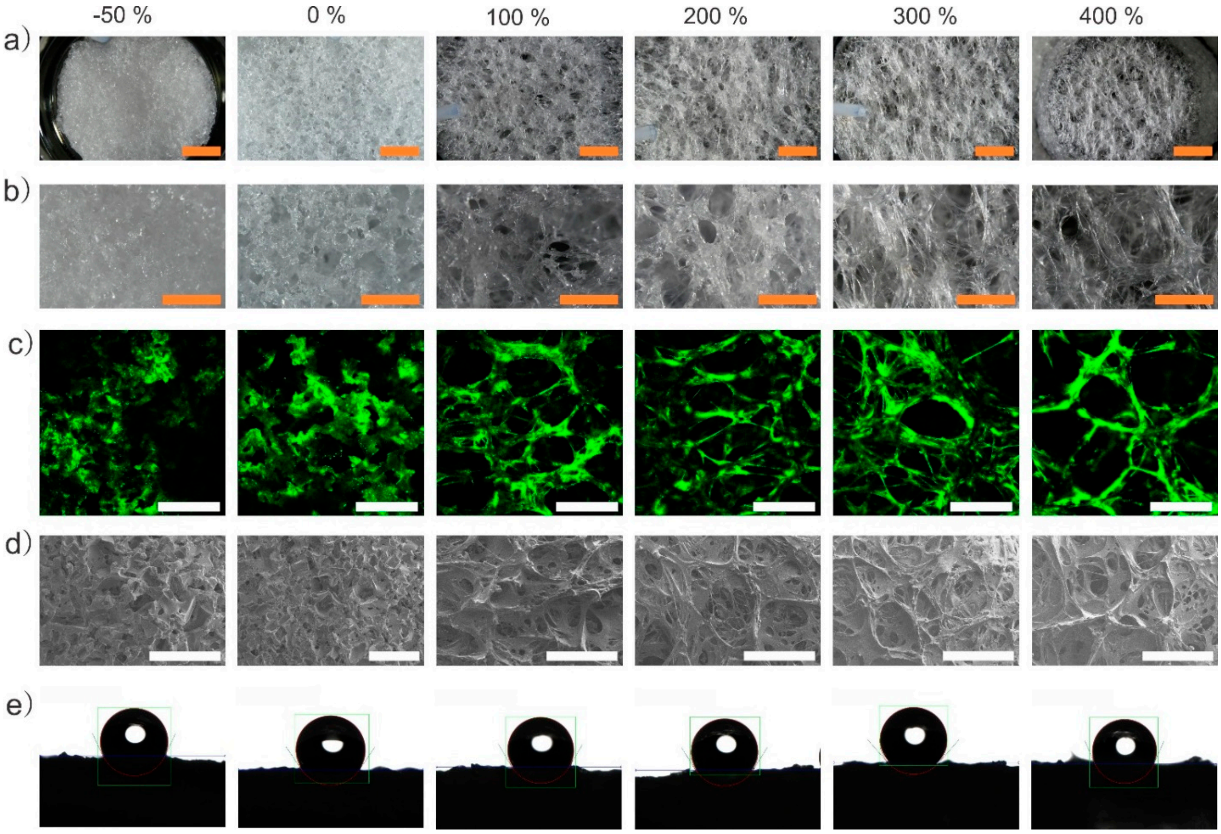

Figure S4 (a) and (b): Microscopy images of the sponge (3.5 mm) at 10× (scale bar 3 mm) and 30× (scale bar 1.5 mm) magnification, respectively; (c) confocal fluorescence images of the PDMS sponge stained with Rhodamine 6G, where the scale bar is 1 mm; (d) SEM micrographs of the PDMS sponge (scale bar 1.5 mm); and (e) images of the water contact angle experiment on the PDMS sponge for tensile strain ranging from −50% to +400%.

To visualize the different layers of the sponge and to determine the pore size, confocal fluorescence microscopy was employed (Figure S1c). Confocal microscopy makes use of the high lateral (up to approx. 180 nm) and axial (up to 500 nm) resolution due to the filtering out-of-focus light. This enables pore detection at different depths inside the sponge (optical slicing). The lateral and axial resolution of the setup utilized in this experiment can be calculated from knowledge of the average wavelength ($\bar{\lambda}$, average of excitation and emission wavelength), the numeric aperture (NA), and the refractive index (n) (Equation 2).[57]

$$d_{lateral,conf}(wave) = \frac{0.37 \cdot \bar{\lambda}}{\mathrm{NA}} \qquad 1$$

$$d_{axial,conf}(wave) = \frac{0.64 \cdot \bar{\lambda}}{\left(n - \sqrt{n^2 - \mathrm{NA}^2}\right)} \qquad 2$$

The lateral and axial resolution were calculated to approx. 1 µm and 15 µm, respectively. This resolution suffices for optical slicing of the big pores of the sponge. The as-made sponge was stained for the confocal fluorescence imaging with Rhodamine 6G in an ethanol solution and subsequently dried using compressed air. The confocal fluorescence images reveal that the mean pore radius is increasing from 35 µm at a tensile strain of −50% (compressed) to 145 µm. This increase appeared to be linear (see Figure S2). However, at a tensile strain of 100%, the measured pore radius was higher than expected, which might be related to an inhomogeneous sample.

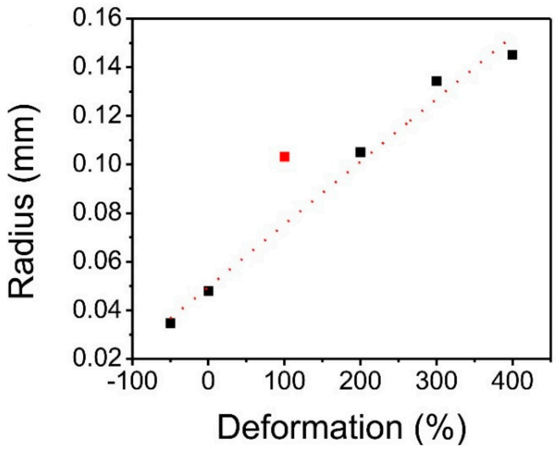

Figure S5 Pore size as determined by CLSM dependent on the tensile strain. The outlier was marked red and not taken into account for the linear fit (red dotted line).

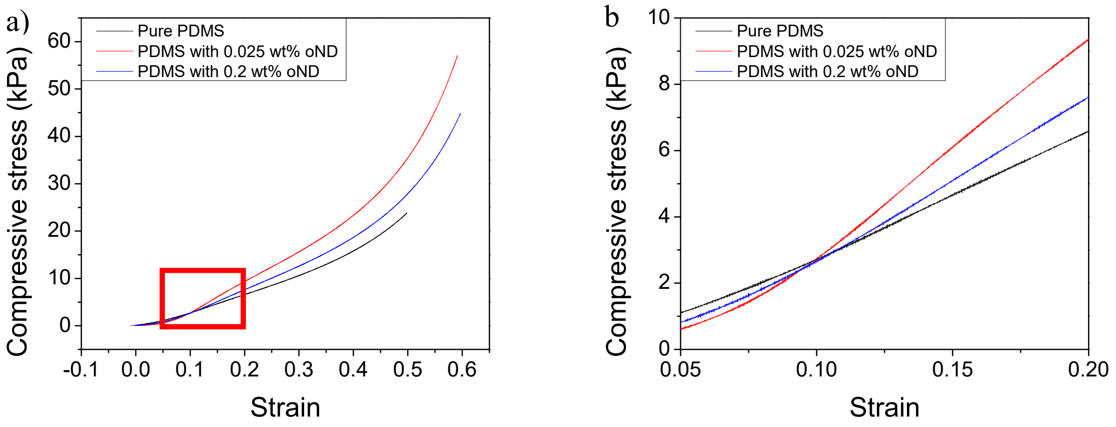

Figure S6 Compressive stress–strain graph for determination of the compressive modulus of PDMS sponges and PDMS/oND composite sponges (a) and its magnification (b) at the location specified with a red rectangle in the graph of (a). The part of the graph in (b) was utilized to determine the compressive modulus of the sponges.

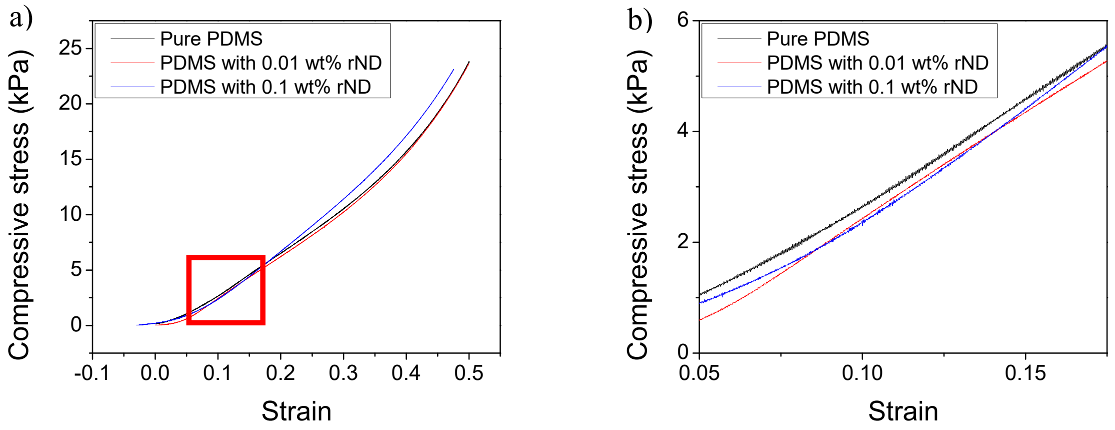

Figure S7 Compressive stress–strain graph for determination of the compressive modulus of PDMS sponges and PDMS/rND composite sponges (a) and its magnification (b) at the location specified with a red rectangle in the graph of (a). The part of the graph in (b) was utilized to determine the compressive modulus of the sponges.

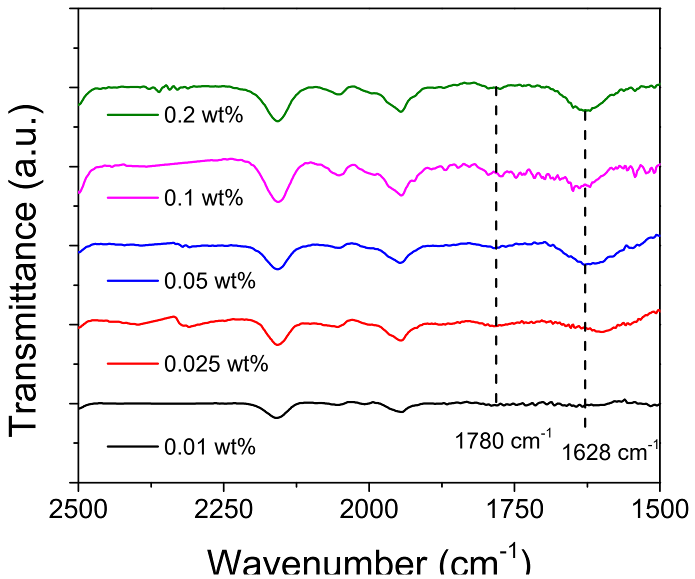

Figure S8 Zoom in of the FTIR spectra (main Manuscript, Figure 4), signifying the change in the absorption bands at 1780 and 1628 cm^{-1}.

Oil/water separation:

A small amount of paraffin oil (approx. 5 ml) was stained with Oil red O. Then, water was added and rigorously stirred to achieve an oil-in-water emulsion. Subsequently, 8 PDMS/rND composite sponges were added to the rigorously stirred emulsion. After 10 min, the oil-soaked sponges were removed and a photo was taken. This process was repeated several times (three times for composite sponges). The separation process was conducted at room temperature (around 22 °C).

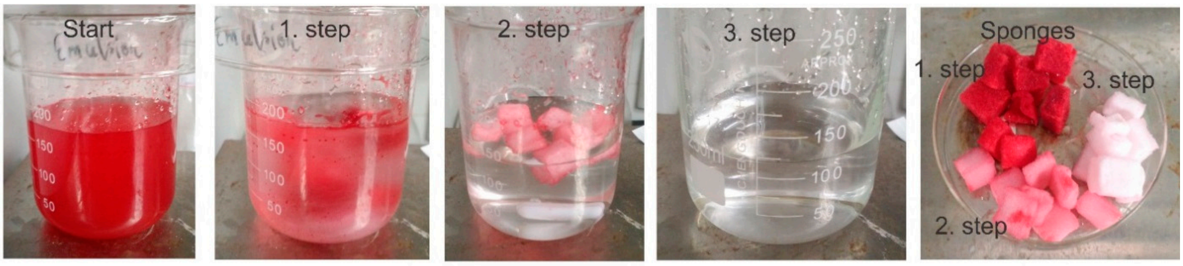

Figure S9 Separation of a paraffin oil/water emulsion in three steps with a PDMS/rND composite (0.025 wt % rND) sponges.

Finally, microscopy images of the “clean” solution were taken to visualize that paraffin microdroplets (here without dye) could be removed by the composite sponges.

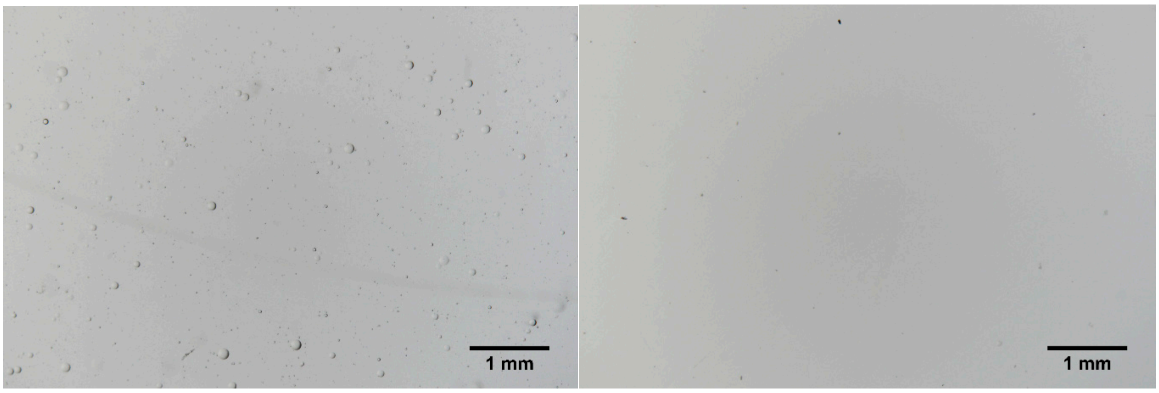

Figure S10. Microscopy images of the (paraffin) oil-in-water emulsion after the 2nd and 3rd cleaning step, as depicted in Figure S9.

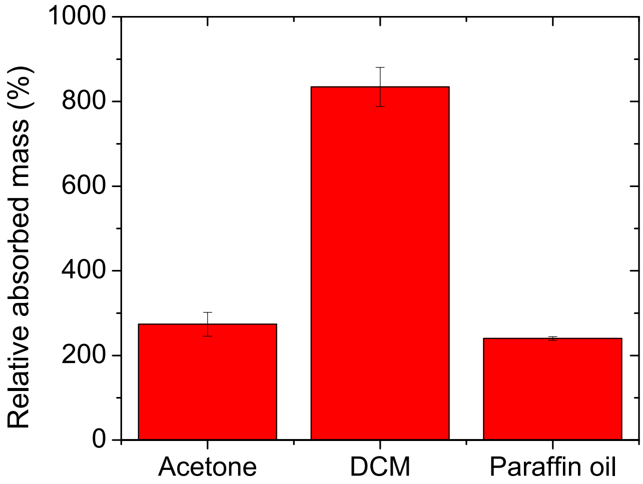

Figure S11. Relative absorbed masses of acetone, dichloromethane, and paraffin oil by the rND-PDMS composite sponges (0.05 wt %).
